# Molecular Characterization of the Multidrug Resistant *Escherichia coli* ST131 Clone

**DOI:** 10.3390/pathogens4030422

**Published:** 2015-06-26

**Authors:** Mark A. Schembri, Nouri L. Ben Zakour, Minh-Duy Phan, Brian M. Forde, Mitchell Stanton-Cook, Scott A. Beatson

**Affiliations:** 1 School of Chemistry & Molecular Biosciences, the University of Queensland, Brisbane, Queensland 4072, Australia; E-Mails: n.benzakour@uq.edu.au (N.L.B.Z.); m.phan1@uq.edu.au (M.-D.P.); b.forde@uq.edu.au (B.M.F.); m.stantoncook@uq.edu.au (M.S.-C.); 2 Australian Infectious Diseases Research Centre, the University of Queensland, Brisbane, Queensland 4072, Australia

**Keywords:** uropathogenic *Escherichia coli*, ST131, urinary tract, FimH, virulence, genomics, TraDIS, plasmid, antibiotic resistance

## Abstract

*Escherichia coli* ST131 is a recently emerged and globally disseminated multidrug resistant clone associated with urinary tract and bloodstream infections in both community and clinical settings. The most common group of ST131 strains are defined by resistance to fluoroquinolones and possession of the type 1 fimbriae *fimH*30 allele. Here we provide an update on our recent work describing the globally epidemiology of ST131. We review the phylogeny of ST131 based on whole genome sequence data and highlight the important role of recombination in the evolution of this clonal lineage. We also summarize our findings on the virulence of the ST131 reference strain EC958, and highlight the use of transposon directed insertion-site sequencing to define genes associated with serum resistance and essential features of its large antibiotic resistance plasmid pEC958.

## 1. Introduction

Uropathogenic *Escherichia coli* (UPEC) are a major cause of urinary tract infections (UTI), causing ~80% of all cases [1]. Over the last few decades, several pandemic clones of UPEC, some of which are associated with multidrug resistant infections, have disseminated worldwide. This includes UPEC clones belonging to several multi-locus sequence types, including sequence type 131 (ST131), ST69, ST73 and ST95 [2,3].

*E. coli* ST131 was originally identified in 2008 as a major clone linked to the spread of the CTX-M-15 extended-spectrum β-lactamase (ESBL)-resistance gene [4,5,6], the most widespread CTX-M ESBL enzyme worldwide [7,8]. ST131 strains have now been identified in both hospital and community settings from virtually all parts of the globe [9,10,11,12]. ST131 causes a variety of extra-intestinal infections, most commonly UTI and bacteremia. Many ST131 strains exhibit resistance to multiple antibiotics, and therefore these infections are often associated with limited treatment options and frequent recurrences. The largest sub-clonal lineage of *E. coli* ST131 is resistant to fluoroquinolones and contains the type 1 fimbriae *fimH*30 (*H*30) allele [13].

Three complete ST131 genome sequences have been generated. This includes SE15 [14], EC958 [15] and JJ1886 [16]. Another ST131 strain, NA114, while listed among the completely sequenced genomes on the NCBI database, remains in draft format [15,17]. This review will present an overview of our recent genomic analysis of ST131 and provide an update on the molecular characterization of the ST131 reference strain EC958.

## 2. Global Epidemiology of ST131

**Figure 1:**
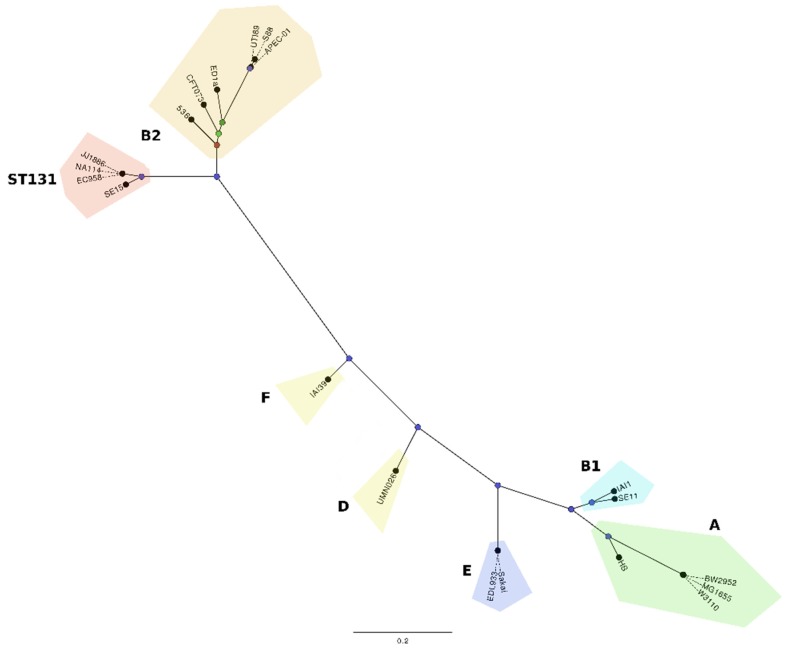
Maximum likelihood phylogenetic comparison of ST131 strains EC958, JJ1886, NA114 (clade C) and SE15 (clade A), and 16 representative strains from other *E. coli* phylogroups. The phylogenetic relationships were inferred with the use of 70,777 SNPs identified between the genomes of the 20 *E. coli* strains and 1000 bootstrap replicates. The major *E. coli* phylogroups are coloured as follows; group B2-ST131: (red); group B2 non-ST131: APEC-01, S88, 536, UTI89, CFT073, ED1A (orange); group D: UMN026, IAI39 (yellow); group A: BW2952, MG1655, W3110, HS (green); group B1: SE11, IAI1 (aquamarine); group E: O157 EDL933, O157 Sakai (blue). Nodes are coloured according to bootstrap support for branching at that node: 1000 (blue), 858 (dark green), 770 (light green), 659 (red). The Figure is adapted from Forde *et al*. 2014 [15].

ST131 belongs to the *E. coli* phylogenetic group B2, which encompasses the largest group of *E. coli* associated with extra-intestinal infections. Based on phylogenetic analyses, the ST131 strains EC958, NA114 and JJ1886 cluster together in a clade discrete from SE15, and separate from representative strains from other *E. coli* phylogroups (Figure 1). Two recent studies have independently examined the global epidemiology of ST131 using genome sequence-based methods [18,19]. These studies identified a globally dominant fluoroquinolone resistant-FimH30 sub-lineage defined as *H*30 [18] or clade C [19]. All strains within this sub-lineage possessed the fluoroquinolone resistance alleles *gyrA*1AB and *parC*1aAB. Further analysis also revealed that ST131 strains containing the *bla*_CTX-M-15_ allele comprised a smaller subset of strains within this sub-lineage and were referred to as *H*30-Rx [18] or clade C2 [19]. Strikingly, the data from both studies supports the recent emergence and global dissemination of this sub-lineage from a single progenitor, provoking intriguing questions with respect to ST131 transmission, colonization and virulence.

In addition to the dominant clade C that comprised 79% of our sequenced ST131 strains, our analysis also identified two other well-supported ST131 clades referred to as A and B [19]. Clade A, represented by the reference strain SE15, was the most divergent and comprised strains that contained the *fimH*41 allele. In contrast, strains from clade B were very similar to those from clade C and characterised by possession of the *fimH*22 allele. The prevalence of these *fimH* alleles, including the dominant *H*30 allele, is consistent with that reported previously from a large and extensive collection of ST131 strains [13].

Our own detailed genomic analysis focused on the major defining features of the three ST131 clades [19]. While sequence analysis did not reveal any significant association with geographic origin, the majority of the single nucleotide polymorphisms that defined each clade were strongly associated with recombination. In total, 137 regions were defined as recombinant within our ST131 strain set, with the majority of large recombinant regions located adjacent to insertion sites for prophages and mobile genetic elements. Other recombination regions within the ST131 strain set were also identified, some of which encompassed virulence genes including *fimH*, the *fliC* flagella major subunit gene, and genes involved in capsule and O antigen biosynthesis. One other notable recombination region encompassed the *fimB* recombinase gene that contributes to the regulation of type 1 fimbriae expression. Most ST131 strains from clade C have a 1,895bp insertion element within the *fimB* gene (*fimB::ISEc55*), suggesting they may possess an altered type 1 fimbriae expression profile. Indeed, the *fimB::ISEc55* insertion has been associated with a slower “off”-to-“on” type 1 fimbriae switching phenotype in ST131 [20,21]. We are currently investing the impact of this insertion on ST131 virulence.

## 3. Molecular Characterisation of the ST131 Reference Strain EC958

EC958 is an O25b:H4 serotype strain isolated in 2005 from the urine of an 8-year old girl presenting with a community-acquired UTI in the United Kingdom [21]. The complete genome sequence of EC958 has been determined [15]. EC958 contains multiple genes associated with UPEC virulence, including genes encoding adhesins (e.g., type 1 fimbriae, curli and the afimbrial adhesin), autotransporter proteins (e.g., Ag43, UpaG, UpaH and PicU) and the biosynthesis of several siderophores (enterobactin, aerobactin and yersiniabactin).

**Figure 2:**
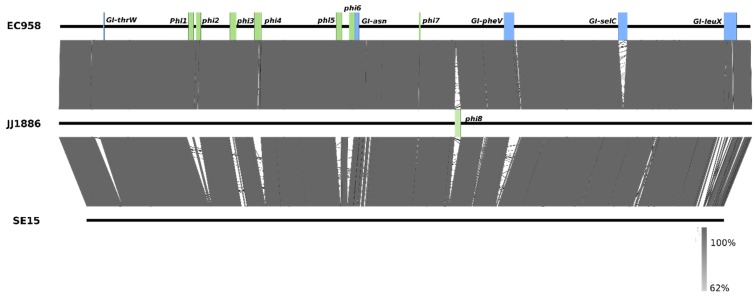
BLASTn pairwise comparison of the genome sequences of EC958, JJ1886 (both clade C) and SE15 (Clade A). Major regions of variation in the structure and location of genomic islands (green) and prophage elements (blue) are indicated. Grey shading indicates nucleotide identity between sequences (62%–100%). Figure prepared using Easyfig [27].

Both EC958 and JJ1886 belong to the globally dominant CTX-M-15 positive, fluoroquinolone resistant, *H*30 clade C ST131 sub-lineage. The two strains display a high level of synteny at the core genome level, with major differences due to the number, content and location of genomic islands (GIs) and other mobile elements (Figure 1). For example, GI-*selC* is present in EC958 but not JJ1886, while the Phi8 prophage is only present in JJ1886. The two strains cluster distinct from the ST131 clade A SE15 strain. Based on whole-genome BLASTn comparisons, the major structural differences between EC958/JJ1886 and SE15 are the presence of seven prophage loci (Phi1-Phi7) and four genomic islands (GI-*thrW*, GI-*pheV*, GI-*selC*, and GI-*leuX*) (Figure 2). Future examination of complete genomes of ST131 strains from different origins will be required to determine the extent of divergence of prophage, genomic islands and other mobile genetic elements in the ST131 clonal group.

## 4. Virulence of *E. coli* ST131

EC958 has been characterised extensively with respect to several virulence characteristics. The strain possesses the *fimB::*IS*Ec55* insertion but can express type 1 fimbriae after several rounds of static subculture. The expression of type 1 fimbriae by EC958 is required for adherence to and invasion of human T24 bladder epithelial cells, and colonization of the mouse bladder [21]. In mice, *E. coli* EC958 causes acute and chronic UTI [22]. EC958 bladder infection involves the formation of intracellular bacterial communities (IBCs) in superficial epithelial cells and the subsequent release of rod-shaped and filamentous bacteria into the bladder lumen [22]. EC958 also causes impairment of rat uterine contractility [23].

The ability of EC958 to resist the bactericidal action of human serum has been extensively interrogated using hyper-saturated transposon mutagenesis in combination with transposon directed insertion-site sequencing (TraDIS) [24]. TraDIS is a high-throughput functional genomics method that enables a pool of transposon mutants to be characterized by direct sequencing of DNA flanking transposon insertion sites [25]. In total, 56 genes were defined by TraDIS to comprise the EC958 serum resistome, of which 46 genes were validated by the generation and testing of specific mutants. The majority of these genes encode outer membrane proteins, or were associated with the biosynthesis of lipopolysaccharide (LPS), the enterobacterial common antigen or colonic acid. Overall, the murein lipoprotein Lpp and two lipidA-core biosynthesis enzymes (WaaP and WaaG) were most strongly associated with serum resistance. The *hyxR* gene, which has previously been shown to contribute to the nitrosative stress response and intramacrophage survival of UPEC [26], was also identified as a minor regulator of O-antigen chain length.

## 5. Plasmids of ST131

**Figure 3:**
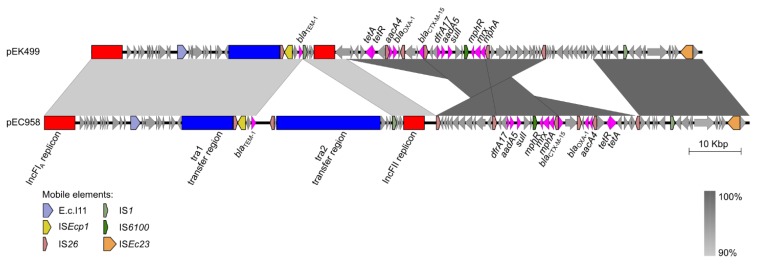
Sequence comparison between two IncF plasmids from ST131 strains; pEC958 [29] and pEK499 [30]. Figure prepared using Easyfig [27].

Plasmids represent a major vehicle for the carriage of antibiotic resistance genes. Among the *Enterobacteriaceae*, plasmids from a range of incompatibility (Inc) groups have been characterised that contain various combinations of resistance, conjugative transfer and other cargo genes. The diversity of plasmid types in ST131 has been examined, with 50% of the most frequent gamma-proteobacterial plasmid groups identified within the ST131 lineage [28]. Our own analysis revealed that the majority of ST131 strains harbor an IncF plasmid, many of which are associated with the carriage of antibiotic resistance genes [29]. Indeed, complete genome sequencing of EC958 demonstrated it contains a large 135.6 kb plasmid that harbors two replicons (RepFIA and RepFII) and 12 antibiotic resistance genes (including *bla*_CTX-M-15_). The most closely related plasmid to pEC958 is pEK499 (99% identity covering 85% of pEC958; Figure 3), which was also isolated from an ST131 strain in the United Kingdom [30]. Interestingly, despite the presence of the *bla*_CTX-M-15_ gene on pEC958, we have shown that this is not the major determinant responsible for EC958 resistance to second and third generation cephalosporins. Instead, EC958 contains a chromosomally-located *bla*_CMY-23_ gene that drives this resistance phenotype [31].

We employed TraDIS as a novel approach to investigate the biology of pEC958 [29]. Analysis of TraDIS data from our saturated transposon mutant library of EC958 identified 27,317 reads that mapped to unique insertion sites in plasmid pEC958 (*i.e.*, one insertion site every 4.96 bp). Genetic elements required for pEC958 stability were identified in both the RepFIA and RepFII replicons; the *ccdA*, *sopA* and *sopB* genes in RepFIA, and the *copA*, *repA6*, *repA1*, *repA4* genes as well as the *oriV* region in RepFII. Interestingly, this data suggests a model where both replicons contain features that ensure their stable inheritance: replication in RepFII and partition as well as post-segregational killing in RepFIA. Our analysis also identified EC958_A0140 as a novel gene of unknown function that is associated with pEC958 stability. Screening of the NCBI complete plasmid sequence database revealed EC958_A0140 is present in 17 other plasmids, all of which are IncF type except for pECL_A (non-typable). However, bioinformatic analysis of EC958_A0140 did not yield any clues regarding its function and thus this remains an area of ongoing study.

## 6. Conclusions

Our current understanding of ST131 epidemiology supports its divergence into three discrete sub-lineages sometime before the year 2000, with acquisition of multiple mobile genetic elements, associated recombination events and point-mutations jointly responsible for the emergence of the most prevalent clade C/*H30* strains. Several studies have now reported the identification of ST131 strains resistant to last-line carbapenem antibiotics [32,33,34,35], highlighting the alarming scenario of pan-resistance in a UPEC clone that has already demonstrated its capacity to disseminate rapidly across the globe. Future work will explore the continued evolution of the globally dominant clade C/*H30* group, and address important questions that relate to ST131 resistance, transmission, colonization and virulence.
